# The Impact of Cardiac Diseases during Pregnancy on Severe Maternal Morbidity and Mortality in Brazil

**DOI:** 10.1371/journal.pone.0144385

**Published:** 2015-12-09

**Authors:** Felipe F. Campanharo, Jose G. Cecatti, Samira M. Haddad, Mary A. Parpinelli, Daniel Born, Maria L. Costa, Rosiane Mattar, Brazilian Network for Surveillance of Severe Maternal Morbidity Study Group

**Affiliations:** 1 Department of Obstetrics, Federal University of São Paulo (UNIFESP), São Paulo, Brazil; 2 Department of Obstetrics and Gynecology, State University of Campinas (UNICAMP) School of Medicine, Campinas, Brazil; 3 Discipline of Cardiology, Federal University of São Paulo (UNIFESP), São Paulo, Brazil; University of Barcelona, SPAIN

## Abstract

**Background:**

To evaluate maternal heart disease as a cause or complicating factor for severe morbidity in the setting of the Brazilian Network for Surveillance of Severe Maternal Morbidity.

**Methods and Findings:**

Secondary data analysis of this multicenter cross-sectional study was implemented in 27 referral obstetric units in Brazil. From July 2009 to June 2010, a prospective surveillance was conducted among all delivery hospitalizations to identify cases of severe maternal morbidity (SMM), including Potentially Life-Threatening Conditions (PLTC) and Maternal Near Miss (MNM), using the new criteria established by the WHO. The variables studied included: sociodemographic characteristics, clinical and obstetric history of the women; perinatal outcome and the occurrence of maternal outcomes (PLTC, MNM, MD) between groups of cardiac and non-cardiac patients. Only heart conditions with hemodynamic impact characterizing severity of maternal morbidity were considered. 9555 women were included in the Network with severe pregnancy-related complications: 770 maternal near miss cases and 140 maternal death cases. A total of 293 (3.6%) cases were related to heart disease and the condition was known before pregnancy in 82.6% of cases. Maternal near miss occurred in 15% of cardiac disease patients (most due to clinical-surgical causes, p<0.001) and 7.7% of non-cardiac patients (hemorrhagic and hypertensive causes, p<0.001). Maternal death occurred in 4.8% of cardiac patients and in 1.2% of non-cardiac patients, respectively.

**Conclusions:**

In this study, heart disease was significantly associated with a higher occurrence of severe maternal outcomes, including maternal death and maternal near miss, among women presenting with any severe maternal morbidity.

## Introduction

About 287,000 maternal deaths occur each year worldwide, and considerable variation exists between high and low/middle-income populations [1]. Maternal mortality (MM) is not only a health indicator but also a social indicator, although there is a current trend to investigate the impact of maternal morbidity on women’s health [2,3]. Maternal death has direct and indirect causes. “Obstetric transition” occurs in regions of intermediate levels of income and development that are beginning to show a trend towards decreasing maternal mortality and a proportional increase in indirect causes. This has gained more relevance even in middle-income countries [4].

Indirect maternal deaths result from conditions existing before pregnancy or recently developed disease unrelated to pregnancy, e.g. heart disease, HIV/AIDS, chronic hypertension, anemia, cerebrovascular disease, infections. A recent data compilation demonstrated that indirect causes accounted for at least one-fourth of all maternal deaths in Brazil [5]. A decrease in maternal mortality constitutes the Millennium Development Goal 5. The target was to decrease maternal mortality ratio (MMR) by 75% from 1990 to 2015. In Brazil, a 51% reduction in MMR has occurred since 1990, however this decline is still insufficient considering that the goal for Brazil is 35/100.000LB until 2015 [6].

Maternal morbidity, a more common condition, also affects women’s lives and is increasingly valued. Then maternal near miss (MNM) has gained importance in the last 20 years [7] and the World Health Organization (WHO) defined it as a condition in which “a woman nearly died, but survived during pregnancy, childbirth or postpartum period, merely by chance or good hospital care” [8].

Cardiac disease encompasses a broad and heterogeneous group of pathological conditions. Heart conditions currently represent the main cause of indirect maternal obstetric deaths in high-income countries [9,10]. Despite a low prevalence rate of heart disease in pregnancy (around 1 to 4%), it significantly increases the risk of adverse perinatal events in both the mother and fetus [11,12].

Pregnancy is associated with substantial and progressive hemodynamic changes starting early in pregnancy, reaching their peak at the end of the second trimester and remaining relatively constant until childbirth. Major alterations in pregnancy include a 30 to 50% increase in both cardiac output and blood volume, in addition to decreased blood pressure. In cardiac pregnant patients, these modifications may lead to clinical decompensation [12,13], exposing these women to potentially life-threatening situations.

Heart disease may be either congenital or acquired. In the past, rheumatic heart disease was the most common condition associated with pregnancy and it continues to predominate in low and middle-income countries. In Brazil, the condition is still the most common cause of heart disease in pregnancy with an estimated proportion of 50% [11,12]. In high-income countries, congenital heart disease predominates, since advances in surgical/anesthetic techniques and cardiac care have increased the survival of these females until fertile age [11,13].

Many factors may delay pregnancy nowadays. Professional stability and desire to attain financial security are some reasons for that. The prevalence of risk factors is increased in the obstetric population, with hypertension, diabetes and dyslipidemia, justifying the increased association between pregnancy and acquired heart disease [10,12]. Maternal-fetal risk is directly related to maternal hemodynamic status [12] and the nature of the preexisting cardiac lesion. Risk ranges from situations where pregnancy is well-tolerated with favorable outcomes to situations of high maternal death risk (25 to 50%) [10]. The importance of heart disease as a risk factor or determinant of maternal near miss events has not yet been fully evaluated. Heart disease of any etiology theoretically increases the occurrence of maternal near miss episodes.

The main aim of the current study was to evaluate heart disease as a cause or complicating factor for severe maternal morbidity and mortality in the setting of the Brazilian Network for Surveillance of Severe Maternal Morbidity. Secondary aims were to compare sociodemographic, obstetric, perinatal characteristics and maternal morbidity features between women with and without heart disease.

## Method

The current study is a secondary data analysis of the Brazilian Network for Surveillance of Severe Maternal Morbidity, focused on the impact of heart disease in the development of severe maternal morbidity. The Network was a multicenter cross-sectional study, implemented in 27 referral obstetric units located in the entire Brazilian territory. During an one-year period, a prospective surveillance of all obstetric hospitalizations was performed to identify cases of PLTC, MNM and MD, using the WHO criteria [8]. Details of the current study methods have already been published elsewhere [14,15].

Sample size calculation was based on 75 thousand deliveries. Surveillance of these deliveries should be carried out to identify approximately 100 maternal deaths and 600 Maternal Near Miss cases (based on a prevalence of 8 MNM cases per 1,000 deliveries [14], a ratio of maternal deaths to live births of 140:100,000 and a 95% confidence interval). A wide geographical distribution of participating centers was achieved, including all Brazilian regions.

The research team in each participating center carried out surveillance and data collection of all hospitalized women meeting the inclusion criteria (presence of severe conditions). Data was retrieved from patient medical charts and information was entered into previously encoded forms, immediately after hospital discharge, transfer or death of the patient. Data was transferred to a specific platform (OpenClinica^®^ version 2.5.5—Waltham, MA, EUA). There was strict quality control of the data, with preparatory meetings for development and training in the use of data collection forms, use of the standard operating procedures manual, technical visits to each center (with random case selection for verification), in addition to systematic data consistency verification by local investigators and study coordinators. Specific inconsistency lists were available for each participating center. Some inconsistencies were logical and the coordinating team only evaluated information on the respective database for correction. For the remaining inconsistencies, a list was sent to each center where local researchers checked medical charts and/or the health professional responsible for the patient to obtain necessary information. Corrections were then entered into the database and new consistencies were run until no more inconsistencies appeared [15].

### Definition of variables

Study variables included sociodemographic characteristics; clinical and obstetric history; details of personal history, occurrence of complications, maternal health care and existence of delays; maternal outcomes recently defined by the WHO—Potentially Life-Threatening Conditions and criteria for Maternal Near Miss (clinical or laboratory evidence of organ dysfunction or failure, and management procedures for severity, including mechanical ventilation, use of vasoactive drugs or hysterectomy due to infection or hemorrhage) [8]. Birth conditions (number of live births, Apgar score at 5 minutes and weight) and neonatal outcome were also used to compare groups.

Inclusion criteria for women with preexisting heart disorder or cardiac disease diagnosed during pregnancy were identical to those of the original study, i.e., only cases presenting any complication classified as a potentially life-threatening condition or maternal near miss event were included. Women suffering from pre-existing heart disease or a cardiac condition identified during pregnancy had one of the conditions listed in Table 1. Conditions were specified in the operating procedures manual of the study [15]. Women with cardiac arrhythmia in normal hearts and normally functioning biologic valve prosthesis were excluded from the study.

**Table 1:** Cardiac conditions included in the Brazilian Network for Surveillance of Severe Maternal Morbidity

• Aortic aneurysm • Non-operated cyanotic congenital heart diseases • Previous acute myocardial infarction • Mitral stenosis with atrial fibrillation • Non-operated congenital heart disease with hemodynamic repercussions (aortic coarctation) • Need for anticoagulation therapy (mechanical prosthesis) • Atrial fibrillation with heart failure and ventricular dysfunction	• Marfan syndrome with aortic involvement • Pulmonary hypertension • Eisenmenger syndrome • Hypertrophic cardiomyopathy • Dilated cardiomyopathy • Congenital heart disease diagnosed as complicated • Severe valvular disease (aortic/mitral stenosis—advanced functional class) • Takayasu's disease • Heart failure class II and III • Peripartum cardiomyopathy

### Data analysis

For the current analysis, women were divided into two groups: those with cardiac conditions and women with the remaining complications. Women still pregnant at the end of study data collection were excluded from analysis. Maternal characteristics were distributed and assessed considering previous conditions: sociodemographic characteristics (age, ethnicity, years of schooling, marital status, BMI), obstetric characteristics, pregnancy outcomes, delivery route and neonatal outcome. Women with and without heart disease were compared and the difference between groups was evaluated by the chi-square test adjusted for clustering design effect.

The prevalence ratios (and their 95% confidence intervals) adjusted for cluster effect for maternal death, maternal near miss, severe maternal outcomes, and potentially life-threatening conditions were calculated and compared between both groups. The Maternal Near Miss Ratio, Severe Maternal Outcomes Ratio (SMOR = MNM + MD), ratio of MNM to MD and Maternal Mortality Ratio were estimated, according to WHO recommendations [8,16].

Major causes related to PLTC (first complication in the chain of events leading to these conditions), the WHO criteria for MNM and perinatal outcomes were comparatively described between both groups. The p-value obtained by the chi-square test, as well as 95% confidence interval values of the prevalence ratio were adjusted for the clustering design effect. Each center was assigned as a cluster and variability in response for each variable had low ICC values [17]. SPSS package (SPSS, Inc., 2009, Chicago, IL, www.spss.com) was the main statistical package used in this analysis.

### Ethical Statement

This study is a secondary analysis. All records were obtained from the database of the main study, the Brazilian Network for Surveillance of Severe Maternal Morbidity. According to rules of the sponsoring agency, the database is not of public domain. The main investigators are the data owners responsible for data used for scientific purposes. We followed all the principles regulating research in humans defined by the Brazilian National Health Council, as well as the Declaration of Helsinki. There was no need for Informed Consent, since data collection was obtained from postmortem medical records or after patients had been discharged from the hospital. There was no contact with the subjects. Local IRBs (listed below) and the National Research Ethics Committee (CONEP, Brazilian Ministry of Health) approved the study, under letter of approval 097/2009.

The Review Boards of the following institutions reviewed and approved this study: Maternidade Cidade Nova Dona Nazarina Daou (Manaus, AM), Maternidade Climério de Oliveira (Salvador, BA), Hospital Geral de Fortaleza (Fortaleza, CE), Hospital Geral Dr. César Cals (Fortaleza, CE), Maternidade Escola Assis Chateaubriand (Fortaleza, CE), Hospital Materno Infantil de Goiânia (Goiânia, GO), Hospital Universitário da Universidade Federal do Maranhão (São Luis, MA), Maternidade Odete Valadares (Belo Horizonte, MG), Instituto de Saúde Elıdio de Almeida (Campina Grande, PB), Hospital Universitário Lauro Wanderley da Universidade Federal da Paraíba (Joao Pessoa, PB), Centro Integrado de Saúde Amaury de Medeiros (Recife, PE), Instituto de Medicina Integral Prof. Fernando Figueira (Recife, PE), Hospital das Clınicas da Universidade Federal de Pernambuco (Recife, PE), Hospital das Clınicas da Universidade Federal do Paraná (Curitiba, PR), Hospital Maternidade Fernando Magalhaes (Rio de Janeiro, RJ), Instituto Fernandes Figueira (Rio de Janeiro, RJ), Hospital das Clinicas da Universidade Federal do Rio Grande do Sul (Porto Alegre, RS), Faculdade de Medicina de Botucatu da Universidade Estadual Paulista (Botucatu, SP), Hospital da Mulher da Universidade Estadual de Campinas (Campinas, SP), Hospital e Maternidade Celso Pierro da Pontifícia Universidade Católica (Campinas, SP), Hospital Israelita Albert Einstein (São Paulo, SP), Faculdade de Medicina de Jundiaí (Jundiaí, SP), Hospital das Clınicas da Faculdade de Medicina de Ribeirão Preto da Universidade de São Paulo (Ribeirão Preto, SP), Santa Casa de Limeira (Limeira, SP), Santa Casa de São Carlos (São Carlos, SP), Casa Maternal Leonor Mendes de Barros (São Paulo, SP), Hospital São Paulo da Universidade Federal de São Paulo (São Paulo, SP).

## Results

**Fig 1:**
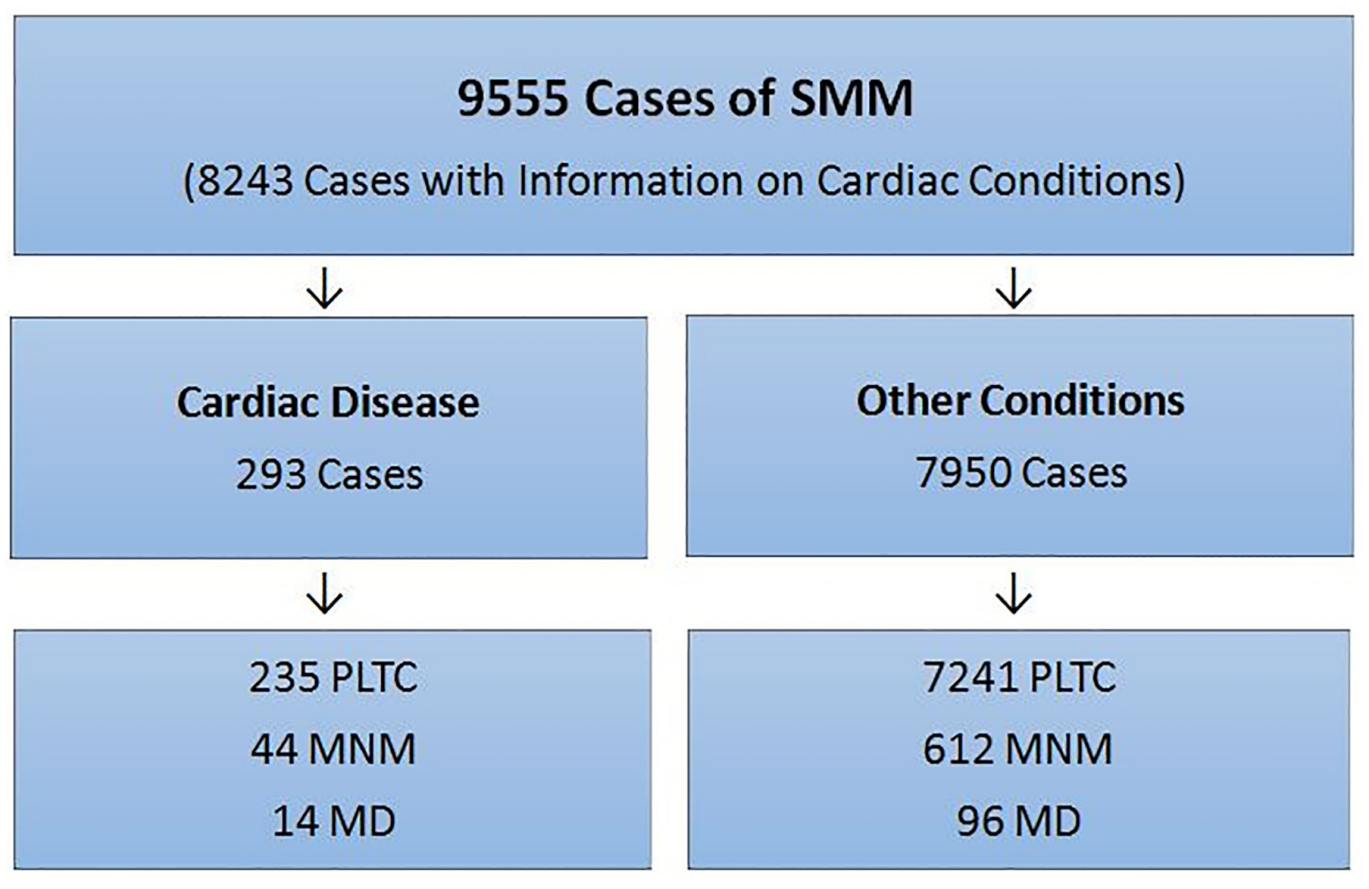
Flow chart of women in the study.

During the one-year study period, 82,388 women were admitted in 27 participating centers, with 82,144 livebirths. Among these women, 9555 had severe pregnancy-related complications and were included in the study. Data on cardiac conditions were available for 8243 of these women. A total of 293 cases (3.1%) involved the presence of heart disease (Fig 1). Of these, 242 (82.6%) had a known heart disorder prior to pregnancy, and 51 (17.4%) had been initially diagnosed during pregnancy. Among the 14 maternal obstetric deaths in cardiac patients, 42.8% (6/14) were diagnosed during pregnancy.

Sociodemographic features differed in groups. A higher frequency of advanced maternal age, a partner and a Body Mass Index indicative of underweight occurred among cardiac patients (Table 2). These women tended to seek prenatal care at the same referral center, had a higher number of previous pregnancies/deliveries and earlier delivery hospitalizations, particularly between 22–33 weeks of gestation. However, gestational age at delivery did not show any statistically significant differences between groups, as was the case also for prenatal care funding, number of previous C-sections/abortion, time since last delivery and route of delivery (Table 3).

**Table 2:** Distribution of women with severe maternal morbidity by sociodemographic characteristics according to the presence of any cardiac disease.

Sociodemographic characteristics	Cardiac Disease		p*
	Yes	No	
**Age (Years)**			
10–19	14.0	17.7	**<0.001**
20–34	58.4	65.3	
35–49	27.6	17.0	
(n)	(293)	(7950)	
**Skin Color**			
White	46.0	43.2	0.592
Others	54.0	56.8	
(n)	(235)	(6419)	
**Schooling (Years)**			
Primary	50.2	46.4	0.531
High School	44.6	47.7	
University	5.2	5.9	
(n)	(211)	(6233)	
**Marital status**			
Partner	65.1	53.6	**0.014**
No Partner	34.9	46.4	
(n)	(249)	(7011)	
**Body Mass Index**			
Underweight	26.4	14.6	**<0.002**
Adequate	35.1	26.9	
Overweight / Obesity	38.5	58.5	
(n)	(148)	(3692)	

* χ^2^ test adjusted for cluster effect

Values in bold mean they are statistically significant.

**Table 3:** Distribution of women with severe maternal morbidity by some obstetric characteristics according to the presence of any cardiac disease.

Obstetric characteristics	Cardiac Disease		p*
	Yes	No	
**Prenatal care**			
Same unit	47.3	23.6	**< 0.001**
Other	49.8	71.4	
No prenatal care	2.8	5.0	
(n)	(283)	(7292)	
**Funding prenatal care**			
Public	93.5	89.0	0.06
Private	3.0	5.0	
No prenatal care	3.4	6.0	
(n)	(263)	(6667)	
**No. of pregnancies**			
1	33.9	41.4	**0.003**
2–3	38.7	38.8	
≥4	27.4	19.8	
(n)	(292)	(7927)	
**No. previous deliveries**			
0	39.7	47.9	**0.002**
1–2	41.1	38.8	
≥3	19.2	13.3	
(n)	(292)	(7927)	
**No. previous abortions**			
0	75.3	77.3	0.311
≥1	24.7	22.7	
(n)	(292)	(7926)	
**No. previous C-sections**			
0	73.1	75.4	0.078
1	21.4	17.1	
≥2	5.5	7.5	
(n)	(290)	(7858)	
**Years since last delivery**			
<2	12.2	10.6	0.73
2–9	68.3	71.1	
≥10	19.5	18.3	
(n)	(123)	(2375)	
**Adequacy of prenatal care**			
Adequate	76.7	75.0	0.602
Inadequate	23.3	25.0	
(n)	(275)	(7629)	
**Gestational age at admission**			
< 22 weeks	8.9	5.4	**0.002**
22–33 weeks	32.6	24.0	
34–36 weeks	19.2	19.6	
≥37 weeks	31.3	46.5	
Postpartum	7.9	4.5	
(n)	(291)	(7761)	
**Onset of labor** ^**#a**^			
Spontaneous	24.9	32.7	0.102
Induced	9.1	10.2	
No labor	61.8	51.6	
Abortion	4.1	5.5	
(n)	(241)	(7311)	
**Gestational age at delivery** ^**#b**^			
< 22 Weeks	3.8	3.6	0.444
22–33 Weeks	23.9	19.0	
34–36 Weeks	20.1	20.9	
≥37 Weeks	52.1	56.5	
(n)	(234)	(7006)	
**Pregnancy outcome/Route of delivery** ^**#c**^			
Vaginal birth	19.3	25.4	0.073
C-Section	76.7	69.2	
Abortion / Ectopic	4.0	5.4	
(n)	(249)	(7369)	

* χ^2^ test adjusted for cluster effect

Values in bold mean they are statistically significant.

The risk of occurring MD, MNM, or Severe Maternal Outcome (SMO = MNM+MD) was estimated to be twofold to almost fourfold higher in cardiac disease patients than in non-cardiac patients. Consequently, the MNM:MD ratio was lower in cardiac patients, meaning that the mortality index increased twofold among these women (Table 4).

**Table 4:** Cardiac disease as the main cause of severe maternal morbidity and mortality and corresponding indicators.

Morbidity/Mortality	Cardiac Disease		PR_adj_ [95% CI]*
	Yes	No	
	n (%)	n (%)	
**Maternal outcome**			
** MD**	14 (4.8)	96 (1.2)	3.7 [2.0–6.9]
** MNM**	44 (15.0)	612 (7.7)	2.0 [1.3–3.2]
** SMO**	58 (19.8)	708 (8.9)	2.2 [1.7–2.8]
** PLTC**	235 (80.2)	7242 (91.1)	0.4 [0.3–0.6]
** Total**	(293)	(7950)	--
**Health Indicators**			
** MNM ratio**	0.5/1000 LB	7.5/1000 LB	
** SMOR**	0.7/1000 LB	8.6/1000 LB	**LB 82,144**
** MNM:MD ratio**	3.1: 1	6.4: 1	
** MMR**	17.0/100,000 LB	115.7/100,000 LB	

* For each group the prevalence ratio, adjusted for cluster effect, was determined for “cardiac disease” versus “non-cardiac disease”.

PR: Prevalence Ratio; CI: Confidence Interval; MD: Maternal Death; MNM: Maternal Near Miss; PLTC: Potentially Life-Threatening Condition; SMOR: Severe Maternal Outcome Ratio; MMR: Maternal Mortality Ratio; LB: Live Birth. Values in bold mean they are statistically significant.

Major causes of PLTC differed significantly between groups. While hypertension and hemorrhage were the most common causes associated with non- cardiac patients, clinical-surgical conditions, e.g. shock, respiratory failure and thromboembolism were significantly more common among cardiac disease patients. The WHO management and laboratory criteria for MNM were similar in both groups. However, clinical criteria were more common in the cardiac group (Table 5).

**Table 5:** Main causes of PLTC and proportion of WHO´s Maternal Near Miss criteria identified among women with severe maternal morbidity according to the presence of any cardiac disease.

Variable	Cardiac Disease		
	Yes	No	p*
**Main causes of PLTC**			
Hypertensive	38.6	71.8	**<0.001**
Hemorrhagic	10.6	23.2	**<0.001**
Infectious	0.7	1.1	0.341
Clinical-Surgical	53.9	9.6	**<0.001**
(n)	(293)	(7950)	
**WHO MNM Criteria**			
Clinical criteria	75.9	54.3	**<0.002**
Laboratorial criteria	55.2	57.3	0.754
Management criteria	63.8	64.9	0.885
(n)	(58)	(707)	--

*χ^2^ test adjusted for cluster effect

Values in bold mean they are statistically significant.

The level of treatment complexity required for both groups was also different. ICU admission was much more common in cardiac patients (52.6%) than in non-cardiac patients (22.6%). Although neonatal death rate almost doubled and the rate of low birthweight was slightely higher among cardiac patients, perinatal outcomes were quite similar between both groups (Table 6).

**Table 6:** Distribution of women with severe maternal morbidity by neonatal outcomes according to the presence of any cardiac disease.

Variable	Cardiac Disease		
	Yes	No	p*
**Neonatal outcome**			
Hospital Discharge	71.1	76.2	0.282
Hospital Admission	23.9	20.6	
Neonatal Death	4.1	2.4	
Transferred	0.9	0.9	
(n)	(218)	(6.382)	
**Condition at birth**			
Live Birth	96.1	95.9	0.887
(n)	(230)	(6.851)	
Apgar 5 Min < 7	8.7	6.2	0.154
(n)	(219)	(6.676)	
Low birth weight <2500g	44.5	37.7	0.225
(n)	(220)	(6.730)	
Weight (g)	2510.9	2667.1	
(IC 95%)	[2.351–2670]	[2527–2807]	

*χ^2^ test adjusted for cluster effect

## Discussion

We performed an analysis of the role of heart disease and its impact on women with severe maternal morbidity evaluated by the Brazilian Network for Surveillance of Severe Maternal Morbidity. Among women with cardiac disease the estimated risk of MNM doubled and that of MD increased almost fourfold, compared with non-cardiac patients. These rates corresponded to the most frequent clinical-surgical causes of MNM and MD. The prevalence rate of pregnancy-related heart disease was 3.1% in severe maternal morbidity cases in this Brazilian study. Heart disease prior to pregnancy was responsible for 82.6% of cases, in disagreement with data in the literature, where most conditions were first diagnosed during pregnancy. This number corresponds to a prevalence rate of 35.6 cases per 10,000 births, which is much higher than data reported for a recent population-based cohort study carried out in the Netherlands [18].

To the best of our knowledge, despite the existence of other prospective studies of heart disorders with corresponding maternal morbidity and mortality [18], this is the first study to use the recent WHO standardized definitions and criteria for different levels of morbidity [8,16]. This is a positive aspect of the current study, which provides standardized results to compare with other findings similarly obtained in the future from other populations.

Pregnancy poses significant challenge to cardiovascular physiology. Previously unknown heart disorders may first become evident during pregnancy, when diagnosis was made in 17.4% of cases in this network. The most common symptoms resembling heart failure (exertional dyspnea, palpitations and edema) are often underestimated or confused with findings that typically occur during normal pregnancy. There may then be a delay and difficulty in making the correct diagnosis [12,19]. In Brazil, as in other parts of the world, maternal mortality due to direct causes has declined in the last decades. In contrast, those due to indirect causes increased. Heart disease is one of the main causes of MM in high-income countries [11]. Emphasis should therefore be placed on heart disease for the development of interventions to reduce maternal morbidity and mortality.

Pregnancy in women over 35 years of age was significantly more common in cardiac patients. The delay in childbirth until later in life and the increased prevalence of risk factors in this population (hypertension, diabetes and dyslipidemia), may explain the association between pregnancy and acquired heart disease among older women, in agreement with other studies [12,18]. There was a significantly higher rate of partners among women with severe complications and heart disease. A partner or other family members can significantly influence a woman’s decison to seek medical care. Nevertheless, there is no clear correlation between these factors, apart from a higher association between older age and higher parity. A clear association between delays and outcome severity has already been reported in the literature, as well as in our original study [20].

Obesity is an aggravating factor for several morbid conditons that occur during pregnancy. However, in the present study this condition was significantly more common among women with complications other than heart disease. Since this variable had a higher proportion of missing data, this represents a limitation of the current study. The cardiac group had higher parity, probably due to increased maternal age. As heart disorder was previously known in the majority of cases, it can thus be assumed that family planning counseling had failed for them [21,22].

Women with heart disorders, especially those with congenital heart disease, tend to become pregnant earlier [23], underestimating the risk for their health condition [24]. Adolescence is a common situation found in 14% of cardiac patients in the study, when ambivalent feelings and equivocal perceptions are much more frequent [25]. Family planning advice is required in these young patients, if strategies are to include a reduction in maternal risk conditions.

Although gestational age at birth did not differ between both groups, at the time of hospitalization it did. Admission was significantly earlier and had a longer duration in cardiac patients. Postpartum hospitalization rates almost doubled in these women, compared to those without heart disease (7.9 X 4.5%, data not shown). The postpartum period is critical, since pregnancy cardiovascular functions change abruptly after birth. Vena cava decompression causes an immediate increase in preload. Autotransfusion by uterine blood volume and redistribution of edema/interstitial fluid with resultant volume overload may lead to pulmonary congestion and heart failure. Some changes, however, may last up to six months [26]. This represents a special group of women at higher risk of complications, even when childbirth occurred without any additional damage to the pre-existing condition. These women deserve postpartum intensive care for early detection and adequate management of potential clinical decompensation.

Despite the already known high rates of Cesarean section among Brazilian women overall population, they are even higher among those with severe maternal morbidity. Vaginal birth is associated with lower blood loss, faster pospartum recovery and lower risk of thromboembolism, while Cesarean section produces more abrupt hemodynamic changes, increasing the risk of postpartum hemorrhage [27]. Although there is insufficient evidence to fully support that Cesarean section best resolves pregnancies with severe maternal complications, it is increasingly common when this condition is present. In the current study, Cesarean section rates were elevated, but still higher among cardiac patients, reaching 77%. In a recent Dutch study, the rate of Cesarean section in the general population was 13%, while in the cardiac disease population it achieved 54.8% [18].

The prevalence ratio of maternal death in the current study was 3.7 times higher in the cardiac group. This was no surprise, since heart disease is the main cause of indirect maternal mortality worldwide. Data reinforce the impact of heart conditions on maternal health. In contrast, the estimated risk of developing Maternal Near Miss in this group doubled, supporting the need for deeper analysis of these survivors to understand health care problems and health-disease processes in this specific population. Although both in Brazil and other emerging countries, heart disease still represents a smaller portion of maternal morbidity and mortality in absolute numbers, its impact becomes clear when the reduced MNM:MD ratio and elevated mortality index are confirmed.

Cardiac decompensation may also worsen perinatal outcomes. Among the conditions potentially determinant of this result are a higher incidence of preterm, low birthweight, fetal growth restriction and low Apgar score. Our study showed that perinatal mortality rates almost doubled among women with cardiac diseases, however this difference was not statistically significant. Preterm birth among cardiac patients, whose previously described rates were around 38% ^18^, affected more than 45% of newborn infants equally in both groups. Similar neonatal outcomes regarding birthweight and Apgar scores were found for both groups, reinforcing what has already been described ^27^. A Swedish population-based study identified among women with cardiac diseases a significantly higher occurrence of lower birthweight, lower gestational length, preterm and small for gestational age for the neonates, however the cardiac condition was due only to congenital heart diseases [28].

Some positive points in this study were a large sample size, rigorous quality control data system and the inclusion of all Brazilian geographical regions. A possible limitation of the study was the lack of heart disease categorization (acquired or congenital, valvular, arrhythmic, ischemic or myocardial injury). Details on the use of specific medications and gestational age at the time of cardiac decompensation were lacking, since this assessment was not part of the aims of the initial Network study. Information was missing in a high proportion of cases for some variables due to retrospective data collection after patient discharge from the hospital. Medical charts had no enough detailed data available in the majority of participating centers.

## Conclusion

In the Brazilian Network for Surveillance of Severe Maternal Morbidity, heart disease was significantly associated with a higher occurrence of maternal near miss events and maternal deaths. Potentially life-threatening conditions differed among groups. Clinical-surgical causes and clinical criteria for maternal near miss events were more common in the cardiac group. In the current study, the majority of complications occurred in cardiac patients with known heart disorders before pregnancy, reinforcing the importance of family planning counseling and specialized prenatal follow-up care in this group.
